# Relative Validity and Reproducibility of a Food Frequency Questionnaire to Assess Energy Intake from Minimally Processed and Ultra-Processed Foods in Young Children

**DOI:** 10.3390/nu11061290

**Published:** 2019-06-07

**Authors:** Louise J. Fangupo, Jillian J. Haszard, Claudia Leong, Anne-Louise M. Heath, Elizabeth A. Fleming, Rachael W. Taylor

**Affiliations:** 1 Department of Medicine, Dunedin School of Medicine, University of Otago, P.O.Box 56, Dunedin 9054, New Zealand; louise.fangupo@otago.ac.nz (L.J.F.); leocl333@student.otago.ac.nz (C.L.); 2 Centre for Biostatistics, University of Otago, P.O.Box 56, Dunedin 9054, New Zealand; jill.haszard@otago.ac.nz; 3 Department of Human Nutrition, University of Otago, P.O.Box 56, Dunedin 9054, New Zealand; anne-louise.heath@otago.ac.nz (A.-L.M.H.); liz.fleming@otago.ac.nz (E.A.F.); 4 Nutrition Society of New Zealand, P.O. Box 2039, Whanganui 4543, New Zealand

**Keywords:** food frequency questionnaire, NOVA, food processing, validity, reproducibility, children, New Zealand, ultra-processed foods

## Abstract

NOVA is a food classification system that categorises food items into one of four categories according to the extent and purpose of their processing: minimally processed food (MPF), processed culinary ingredient (PCI), processed food (PF), or ultra-processed food (UPF). The aim of this study was to determine the relative validity and reproducibility of a food frequency questionnaire (EAT5 FFQ) for measuring daily energy intake (EI kJ) and percentage of daily energy intake (EI%) from each NOVA group in New Zealand children. One hundred parents of five year old children completed the 123 item EAT5 FFQ on two occasions four weeks apart. A 3 day weighed diet record (WDR) was completed on non-consecutive randomly assigned days between FFQ appointments. The FFQ overestimated EI (both as kJ and %) from MPF and UPF, and underestimated intakes from PCI and PF, compared with the WDR. Bland–Altman plots indicated reasonably consistent agreement between FFQ and WDR for MPF and UPF but not PCI or PF. Correlation coefficients between the FFQ and WDR were acceptable for EI (%) for MPF (*r* = 0.31) and UPF (*r* = 0.30). The FFQ differentiated between the highest and lowest quartiles for EI (%) from MPF and UPF foods (*p*-values for the trends were 0.006 and 0.009 respectively), and for EI (kJ) from UPF foods (*p*-value for trend 0.003). Bland–Altman plots indicated consistent agreement between repeat administrations of FFQ for MPF and UPF only, while intra-class correlations suggested good reproducibility for EI (kJ and %) for all four NOVA categories (range 0.51–0.76). The EAT5 FFQ has acceptable relative validity for ranking EI (%) from MPF and UPF. It has good reproducibility for measuring EI from all four NOVA categories, in young children.

## 1. Introduction

In recent years, the NOVA (not an acronym) food classification system has increasingly been used in the literature to categorise foods according to the extent and purpose of food processing. In this system, individual foods and drinks are placed into one of four categories: minimally processed (including unprocessed) (MPF), processed culinary ingredient (PCI), processed food (PF), or ultra-processed food (UPF) [1]. Ultra-processed foods in particular have come under scrutiny. They have been described as “formulations of mostly cheap industrial sources of dietary energy and nutrients plus additives” [2]. In many countries, both their availability and consumption has increased substantially [3,4,5], which is concerning because high UPF consumption has been shown to be predictive of poor diet quality [6,7,8]. 

Large population-level surveys have indicated that UPF now accounts for 30%–60% of total daily energy intake among individuals in France [9], the United Kingdom [10], Brazil [11], Mexico [12], and USA [13]. Studies in young (0–8 years old) children have indicated that 32%–40% of the total energy intake in this age group now comes from UPF [14,15,16,17]. However, most of the studies that have included children have been undertaken in Brazil [14,15,16,17], a middle income country where UPF intakes, while increasing, are lower than those seen in high income countries such as Canada and New Zealand [3]. Diet records [10,11,16] and 24 hour recalls [9,12,13,15,17] have commonly been used to measure the intake of foods from each of the NOVA classification groups. Food frequency questionnaires (FFQs) [14] have been used less frequently, but are a convenient way to estimate usual intake of specific foods, due to their low respondent burden [18]. They should ideally be used in the country where they were developed [19], and be validated to ensure that they adequately measure the intake of foods or nutrients of interest in that population [20]. To date, no FFQ has been validated for its ability to measure total daily energy intake, whether expressed in absolute (kJ) or relative (%) amounts, from the four NOVA categories in primary school-aged children. Therefore, the aim of this paper was to assess the relative validity and reproducibility of the EAT5 FFQ for assessing energy intake from the four NOVA categories, in 5 year old New Zealand children. 

## 2. Materials and Methods 

### 2.1. Study Design

The EAT5 food frequency questionnaire (FFQ) has recently been shown to be a valid and reliable measure of intake of nutrients and food groups of relevance to the gut microbiota [21]. The current analysis used the existing EAT5 FFQ, 3 day weighed diet record (WDR), sociodemographic questionnaire, and anthropometric data from this previous study to validate the EAT5 FFQ for measuring energy intake from each of the four NOVA categories (MPF, PCI, PF, and UPF). Parents and children attended two appointments four weeks apart. At the first appointment, the EAT5 FFQ and sociodemographic questionnaire were completed, and anthropometric measurements were obtained. A 3 day WDR was collected over the next month. The EAT5 FFQ was then administered again at the second appointment. 

### 2.2. Participants

As described elsewhere [21], a convenience sample of 100 parent-child pairs was recruited from Dunedin, Auckland and Wellington (New Zealand) from February 2015 to December 2017. Healthy children aged ≥5.0 to ≤6.0 years were eligible. The Human Ethics Committee of the University of Otago, Dunedin, New Zealand, granted ethical approval for the study (reference number H14/154) and written informed consent was obtained from all parents and children. 

Parents indicated their child’s age, sex, ethnicity, and number of siblings. Each participant’s home address was used to determine their level of household deprivation using the NZDep2013 Index of Deprivation score (range 1–10, where 1 represents the least deprived 10% of New Zealand households, and 10 represents the most deprived) [22]. 

### 2.3. EAT5 Food Frequency Questionnaire

The EAT5 FFQ is a quantitative, 123 item FFQ with 10 frequency-response options: (i) not eaten this month, (ii) less than once a week, (iii) once a week, (iv) two times per week, (v) three times per week, (vi) four times per week, (vii) five times per week, (viii) six times per week, (ix) every day, and (x) if more than once a day—‘how many times per day?’. It is a modified version of the validated EAT FFQ aiming to assess intake in 12–24 month old children [23,24] which was originally adapted from the Southampton Women’s Survey FFQ for infants [25]. Food items are grouped under 11 section headings: (i) bread, crackers and breakfast cereals (*n* = 15), (ii) rice and pasta (*n* = 6), (iii) fruit (*n* = 14), (iv) vegetables (*n* = 21), (v) meat, chicken, fish, eggs, beans (*n* = 17) (vi) spreads (*n* = 4), (vii) cakes, biscuits, snacks (*n* = 11), (viii) milk and dairy products (*n* = 15), (ix) puddings (*n* = 3), (x) drinks (*n* = 7), and (xi) takeaways (*n* = 10). The EAT5 FFQ is intended to be administered by an interviewer to the child’s primary caregiver. Where relevant, natural portion sizes (e.g., ‘1 pear’, ‘1 egg’) are used to describe quantities consumed. Where natural portion sizes do not exist (e.g., for spreads, most vegetables, most breakfast cereals, rice, and pasta), caregivers are asked to demonstrate food volumes with dried beans and rice on plates, bowls, and cups, and these were converted to weights using known data on food density. The EAT5 FFQ is included as supplementary material (Table S1).

### 2.4. Weighed Diet Record (WDR)

Parents completed 3 day WDRs for their children on 3 randomly assigned, non-consecutive days (1 weekend day and 2 week days) over four weeks. They were provided with detailed verbal and written instructions and a calibrated electronic kitchen scale (Salter Vista, Kent, UK ±1 g) at the first visit and then contacted during the collection period so that they could ask further questions. On the second visit, the WDR was collected and checked by trained staff. Diet records were analyzed with the Kai-culator nutritional software package version 1.16a (Department of Human Nutrition, University of Otago, New Zealand) using the nutrient database FOODfiles 2014 [26]. 

### 2.5. NOVA Food Processing Categories

**Figure 1:**
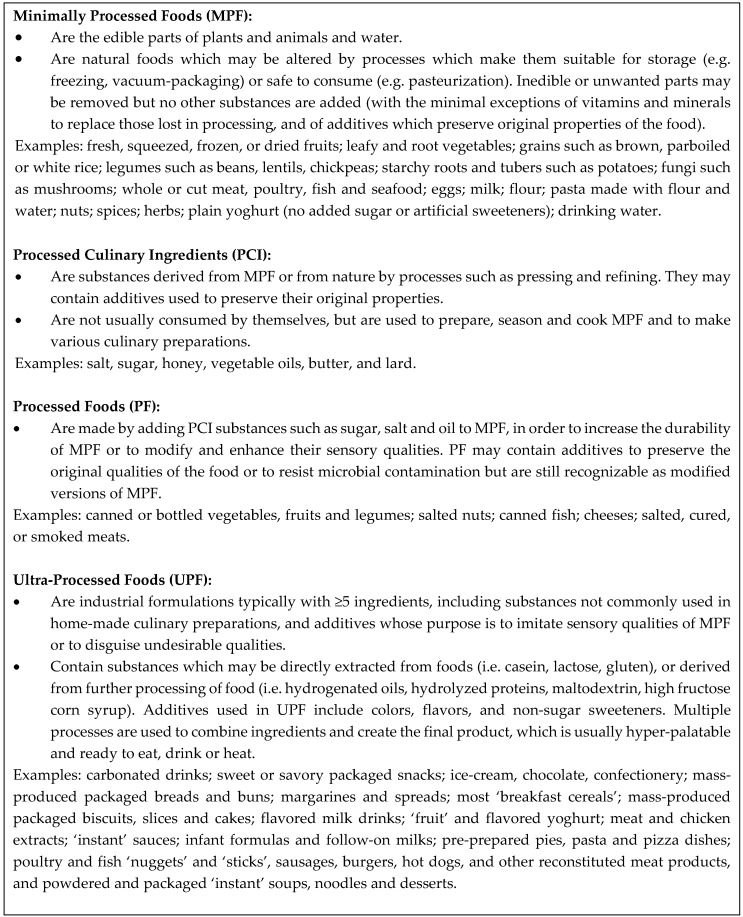
Criteria used to classify food frequency questionnaire (FFQ) and weighted diet record (WDR) foods into NOVA categories of food processing, developed from the work of Monteiro et al. [1,2].

Criteria for NOVA categorization (Figure 1) were applied to all foods in the EAT5 FFQ and the 3 day WDRs. Of the 123 items in the EAT5 FFQ, 99 (80.5%) could be classified wholly into one of the four NOVA categories of MPF, PCI, PF or UPF. The remaining 24 (19.5%) items could not be placed in a single group, mostly because they incorporated foods from more than one NOVA category (e.g. ‘Pears—fresh and canned’, ‘Yoghurt or dairy food’, ‘Subway sandwich’). The majority (*n* = 14) of these items were able to be disaggregated across NOVA groups using weighting criteria for individual ingredients in composite foods established during the development of the FFQ. The nutrient lines associated with multiple foods that were combined in one FFQ item (e.g., “Pears—fresh and canned”) were determined based on previous consumption data in young children or relative market share in New Zealand [23]. The researchers met to reach a consensus regarding categorization of the few remaining foods (*n* = 10).

Of the 1400 different food items in the 3 day WDR, 885 (63.2%) were able to be immediately classified wholly into one NOVA group. The remaining 515 (36.8%) items needed to be disaggregated because they contained food or ingredients from two or more NOVA categories. Of these, 324 (23%) were homemade items for which study participants had provided recipes. The ingredients in each recipe were separated by weight into the four NOVA groups, with weight acting as a proxy for energy content in these cases. A further 110 items were considered highly likely to be homemade although the study participant had not supplied the recipe, and therefore standard recipes from the Kai-culator were used to separate ingredients into the four NOVA categories, again by weight. The remaining 81 items were MPF or PF which had been cooked in a PCI (i.e., “egg, fried in vegetable oil”, “chicken, baked, with oil”) and were also able to be separated using Kai-culator recipes.

### 2.6. Statistical Analysis

For each food item, the proportions assigned to each of the NOVA categories were multiplied by the total energy provided by that food. For each person, daily averages of energy intake and % energy intake for each of the NOVA categories were calculated. For the diet records, daily averages were found using the Multiple Source Method for estimating usual dietary intake [27]. To assess relative validity, the first administration of the FFQ was compared against the diet record for both energy intake (kJ) and % energy intake, for all four of the NOVA categories.

Bland–Altman plots were generated and visually assessed for agreement. Limits of agreement (95%) were also calculated. Mean differences between the FFQ and the WDR in energy intake (and % energy intake) from each NOVA category were calculated with paired t-tests. Confidence intervals, *p*-values, and intra-class correlations between measures were reported. To assess the ranking ability of the FFQ, energy intakes from the FFQ were categorized into quartiles and mean (SD) energy intakes from the diet records for each quartile were calculated. A test for trend was carried out using a regression model with energy intake from the diet record as the outcome variable and energy intake quartiles as the predictor variable. Cross-classification was also investigated by categorizing energy intake by FFQ and diet record into quartiles and assessing the proportion who were correctly classified (same quartile), correctly or adjacently classified (same or next quartile), correctly classified to extreme quartiles (lowest or highest quartile), and grossly misclassified (highest quartile by FFQ and lowest by WDR, or vice versa).

To assess test–retest reliability, the first and second administration of the FFQ were compared. Mean differences, 95% confidence intervals, and *p*-values were calculated with paired t-tests. Intra-class correlation coefficients were calculated using a two-way mixed effects model.

## 3. Results

### 3.1. Study Population

One hundred participants were recruited, of whom 99 parent–child pairs completed the two FFQs and the 3 day WDR. One parent-child pair completed only the first FFQ and the 3 day WDR, meaning that 100 participants were included in the validity analysis and 99 participants in the reproducibility analysis. The 100 young children (44% male) had a mean (range) age of 5.5 (4.9–6.0) years. The participants were mainly of New Zealand European ethnicity (80%), with 13% identifying as Māori. According to the NZDep2013 Index of Deprivation [22], 19% of the participants were from households in the three most deprived deciles (compared to the expected 30% nationally).

### 3.2. Relative Validity and Reproducibility of Energy Intakes (kJ and %) from NOVA Groups

**Figure 2:**
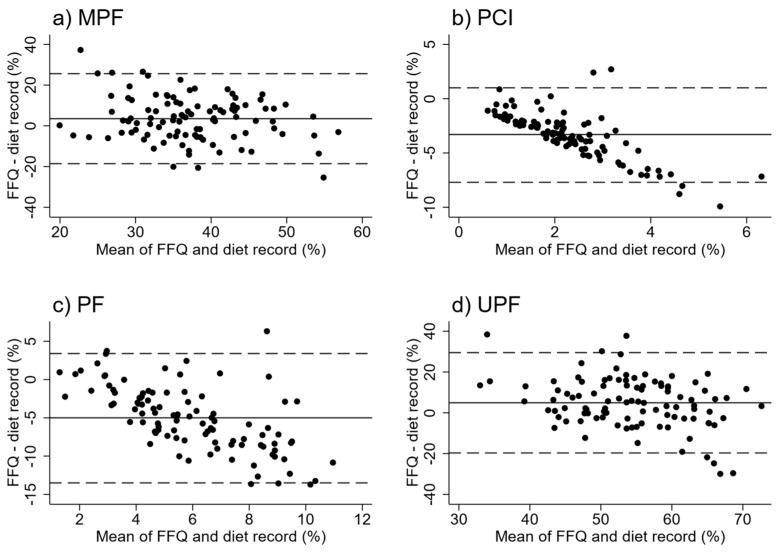
Bland–Altman plots for % energy intake between the FFQ and WDR: (**a**) Minimally Processed Foods (MPF), (**b**) Processed Culinary Ingredients (PCI), (**c**) Processed Foods (PF), (**d**) Ultra Processed Foods (UPF). Dashed lines represent 95% limits of agreement.

Bland–Altman plots indicated reasonably consistent agreement between FFQ and WDR for UPF and MPF but indicated poor agreement for PF and PCI, with the FFQ underestimating intakes at higher intakes (Figure 2). Mean difference in energy intake and percentage of energy intake between the FFQ and WDR were significant for all four NOVA groups, with the FFQ overestimating EI (kJ) and EI (%) from MPF and UPF items, and under-estimating intake from the PCI and PF groups (Table 1). Intra-class correlation coefficients between the FFQ and WDR were acceptable for EI (%) from MPF (*r* = 0.31) and UPF (*r* = 0.30) but were low for all other groups.

The FFQ was able to differentiate between the lowest and highest quartiles for daily EI from UPF (*p*-values for trend 0.003 and 0.009 for kJ and % respectively) and for EI (%) from MPF (*p*-value for trend 0.006) (Table 2). Although three of the eight processed food and energy categories were close to chance (25%), all of the processed food categories had EI kJ or EI % correctly classified into quartiles by the EAT5 FFQ and WDR at levels greater than chance (Table 3). For EI (kJ), correct classification ranged from 23% (MPF) to 31% (PCI), and for EI (%), correct classification ranged from 29% (PF) to 33% (MPF). 

**Figure 3:**
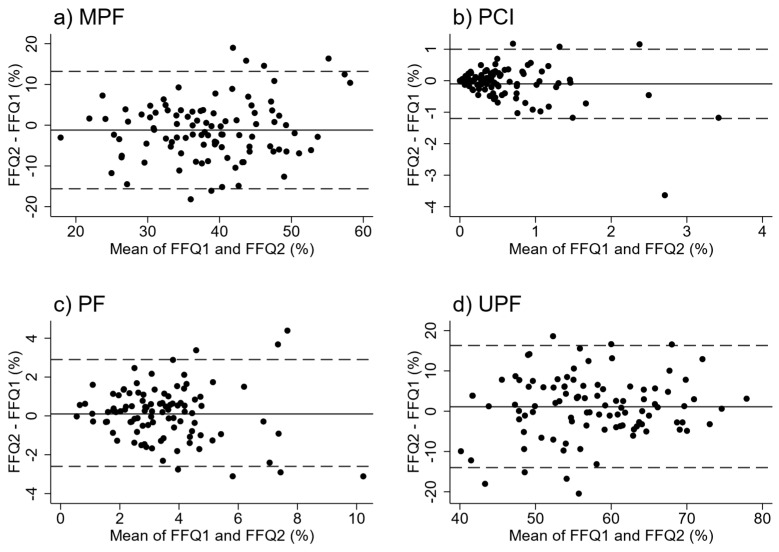
Bland–Altman plots for % energy intake between first and second administrations of FFQ: (**a**) Minimally Processed Foods (MPF), (**b**) Processed Culinary Ingredients (PCI), (**c**) Processed Foods (PF), (**d**) Ultra Processed Foods (UPF). Dashed lines indicate 95% limits of agreement.

Bland–Altman plots indicated consistent agreement between administrations of the FFQ across the range of intakes for UPF and MPF (Figure 3). The mean differences between repeat administrations of the FFQ were not significantly different for EI (%) for any of the four NOVA groups, and MPF was the only group where a significant difference was observed for EI in kJ (*p* = 0.001) (Table 4). Intra-class correlations between repeat administrations of the FFQ suggested good to very good reproducibility for EI as kJ (range 0.51–0.76) and EI as % (range 0.64–0.70) from all NOVA food groups.

## 4. Discussion

We have shown that our EAT5 FFQ provides a valid and reliable tool for ranking EI (%) from MPF and UPF foods, UPF foods being the food group of most interest in this subject area. Although the FFQ overestimated energy intake from these major contributors, the Bland–Altman plots indicated consistent agreement across the range of intakes. However, the FFQ was not able to accurately estimate the EI (kJ) intake from any of the NOVA groups. It was also not able to estimate the EI (%) contribution to the diet from PCI and PF, although these generally represent a much lower proportion of energy intake. 

We cannot compare our findings with the literature as we are not aware of any previous FFQ validation for the measurement of energy intake from NOVA food groups in children, despite at least one FFQ being used to measure intake of ultra-processed foods [14]. Our study appears to be the first to validate an FFQ to measure energy intake from the four NOVA categories of food processing in young children. As concern about the potential health, social, economic, political, and environmental consequences of the increasing consumption of UPF foods has been expressed [2], it is useful to have a way of assessing their intake among school-aged children, who may be exposed to these effects throughout their lives. There are several possible explanations for the lack of agreement for the minor NOVA groups. As defined by Monteiro et al., PCIs such as oils, sugar, and salt, are derived from MPF or from nature by processes such as pressing, refining, and grinding [1,2]. They are rarely consumed alone, with NOVA authors intending that PCIs be used to prepare, season and cook MPF foods to create freshly prepared dishes. With the exceptions of butter and honey, no PCIs were included in the EAT5 FFQ, but they appeared quite frequently in the WDR data, particularly when participants provided recipes which were disaggregated into NOVA groups. This point of difference explains the lack of correlation between PCI in the EAT5 FFQ and WDR. Next, PF are essentially made by adding salt, sugar, oil, or other substances from PCI to MPF, usually have two or three ingredients, and are recognizable as modified versions of MPF such as canned vegetables, cheeses, and fruit in syrup [1.2]. In WDR, these items also often showed up as a proportion of a recipe (e.g., cheese in a child’s sandwich or tinned tomatoes in a mince dish) where the recipes might differ for the same type of food between individual children. By comparison, in the FFQ, PF were often estimated as a percentage of a food item based on average intake of these foods in young children [23]. These methodological differences are likely to have contributed to the lack of agreement between the FFQ and WDR for PF. Overall, we suggest that MPF and UPF are generally straightforward to classify, whereas PCI and PF can be more problematic. 

We chose to disaggregate recipes and composite dishes and apply NOVA classifications at an ingredient level due to the generally high level of detail provided in the WDR. Other groups investigating the intake of UPF in young children have usually classified foods and beverages directly into one group [14,15,16,17] or have undertaken a process whereby composite dishes are disaggregated by ingredient and the composite dish is then classified into the NOVA category which is most predominant among its individual ingredients [28]. Disaggregation has been used in varying degrees in work in cohorts with a wider age range [9,10,12,16]. We propose that our method of disaggregating composite foods by ingredients and consigning each ingredient to a NOVA group is likely to provide a more accurate representation of energy intakes from each of the NOVA groups, than that obtained when composite dishes are consigned to a single NOVA group.

The EAT5 FFQ was specifically designed, and has been validated for, its ability to measure intakes of nutrients and food groups relevant to the gut microbiota (specifically, dietary fiber, soluble and insoluble non-starch polysaccharides) [21]. In that work, the mean correlations between the randomly chosen FFQ and WDR were 0.34 (range 0.24–0.38) for nutrients, and 0.41 (range 0.28–0.56) for food groups, which are higher than the correlations observed for EI (%) from UPF (0.30) and MPF (0.31) in our work. Reproducibility was higher in the validation for nutrients and food groups (*r* = 0.83 and 0.80) than in our validation of NOVA groups (average 0.66) while gross misclassification was lower (average 6% compared to 11% for EI (kJ) and 8% for EI (%)). Overall, we suggest that in order to achieve acceptable validity [20] and even greater reproducibility for all NOVA groups in the future, it would be advisable for researchers to either adapt the EAT5 FFQ, or develop a new FFQ specifically to measure intake of foods with various degrees of processing. The food items in a new or adapted FFQ should each be pre-categorized into a single NOVA group, to avoid the need to disaggregate FFQ items across NOVA groups. However, such an approach would be likely to result in a considerable increase in the total number of food items listed in the FFQ. For example, if adapting the EAT5 FFQ, many foods currently encompassed by one FFQ item would need to be separated into multiple items for accurate NOVA classification (e.g., ‘Pears, fresh and canned’ would need to become ‘Fresh pears’ and ‘Canned pears’). Substantially increasing the number of FFQ items could lead to participant fatigue and thus produce inaccurate results [19]. 

The strengths of our study include the fact that both reliability and validity were assessed, the sample size was adequate for a validation study, and the FFQ was specifically designed for use in New Zealand children. Our study also has some limitations. First, there is likely to have been some misclassification of foods between the four NOVA categories. In some cases it was unclear whether a food was homemade or commercially manufactured. In order to ensure consistency, one researcher (LF) took the lead role in categorizing all foods, using all available information (product name, brand name and/or ingredients if applicable) to decide whether the food was likely to have been homemade (and the recipe thus disaggregated between NOVA groups) or commercial (and thus more likely to fit the UPF criteria). If categorization was still unclear, the researcher consulted other food composition experts until a consensus was reached. Difficulties with classification are not unique to our study; other authors have recognized that the broad NOVA definitions require some interpretation by researchers [29], which means that foods are not always uniformly categorized among different studies [30]. In Australia, thousands of foods within the Australian national food composition database AUSNUT have been categorized according to the NOVA classification [31], thus enabling consistent classification between researchers in that country. However, it is not always possible to determine the brand of food analysed in that database, or to know how similar, or different, Australian food stuffs are in comparison to those from New Zealand. While New Zealand has its own food composition database, to date NOVA classification of the foods within it has not been undertaken. We, therefore, carried out our own classification process so that we could classify foods based on the brand names provided in our New Zealand WDRs and their ingredient lists, which were available online or in local supermarkets. This was also helpful when disaggregating recipes, which sometimes contained specific brands of various ingredients. The use of our own classification in the absence of the uniform coding of the New Zealand food composition database is likely to have provided more accurate results than would have been obtained by using a food database from another country.

Second, we used ingredient weight as a proxy for energy content when recipes were disaggregated across NOVA categories. Weight was used rather than energy content per se because it was a more accessible measure than energy content for individual ingredients. However, as a check, we compared the proportions of NOVA groups in ten different recipes, by energy content (kJ) and weight (g) and found the mean kJ and g intakes to be remarkably similar, as outlined in Table A1.

Limitations relevant to the study population, as described by Leong et al [21], also apply to this work. The study population was not representative of the whole New Zealand population, with a higher proportion of New Zealand Europeans and people from the lower and middle deciles of household deprivation. The FFQ was administered only to the primary caregiver, and as the children were school-aged, they would not have been with their parents at all times. To reduce any resulting omissions, parents were asked to report any food which was provided to the child by another person, and the child attended the appointment with their parent so they could be asked for clarification. 

## 5. Conclusions

The EAT5 FFQ is able to rank EI (%) energy intake from MPF and UPF in young school-age children. It has good reproducibility for measuring energy intake from all four NOVA groups in young children. However, it cannot be used to estimate EI (%) from PCI or PF, or to estimate EI (kJ) from any of the four NOVA groups. An FFQ which is specifically designed to measure energy intake from NOVA groups may be required if intake from all four groups is to be accurately assessed in the future. 

## Figures and Tables

**Table 1:** Difference and intra-class correlations between daily energy intake estimates from processed food categories between FFQ and 3 day WDR (*n* = 100).

	Mean (SD) from WDR	Mean (SD) from FFQ	Mean difference (95% CI) between FFQ and WDR	p-Value	95% Limits of Agreement	Intra-Class Correlation Coefficient between FFQ and WDR
**MPF**						
EI (kJ)	2088 (705)	2798 (967)	710 (503, 918)	<0.001	0 to 2800	0.24
EI (%)	35.6 (10.4)	39.1 (8.3)	3.5 (1.3, 5.7)	0.002	−18.6 to 25.6	0.31
**PCI**						
EI (kJ)	229 (116)	44 (58)	−185 (−211, −158)	<0.001	−452 to 82	−0.05
EI (%)	4.0 (2.0)	0.6 (0.7)	−3.3 (−3.8, −2.9)	<0.001	−7.7 to 0.1	−0.01
**PF**						
EI (kJ)	499 (264)	242 (141)	−257 (−318, −196)	<0.001	−870 to 355	−0.05
EI (%)	8.5 (4.0)	3.4 (1.9)	−5.0 (−5.9, −4.2)	<0.001	−13.5 to 3.4	0.07
**UPF**						
EI (kJ)	3009 (820)	4102 (1386)	1093 (818, 1368)	<0.001	0 to 3864	0.26
EI (%)	52.0 (11.9)	56.9 (8.6)	4.9 (2.5, 7.3)	<0.001	−19.7 to 29.5	0.30

Abbreviations: EI, energy intake; FFQ, food frequency questionnaire; kJ, kilojoules; MPF, minimally processed food; PCI, processed culinary ingredient; PF, processed food; SD, standard deviation; UPF, ultra-processed food; WDR, weighed diet record.

**Table 2:** Ranking ability (by quartiles) of the FFQ for daily energy intake from processed food categories (*n* = 100).

	Quartiles of Daily Energy Intake from Processed Food Categories Using Food Frequency Questionnaire				*p*-Value For Trend
**Mean (SD) daily EI from NOVA categories using WDR**	**Quartile 1**	**Quartile 2**	**Quartile 3**	**Quartile 4**	
**MPF**					
EI (kJ)	1882 (427)	2149 (535)	2155 (732)	2166 (992)	0.175
EI (%)	33.3 (7.3)	33.9 (9.3)	33.1 (12.7)	42.1 (9.1)	0.006
**PCI**					
EI (kJ)	207 (97)	228 (123)	263 (114)	217 (129)	0.548
EI (%)	3.1 (1.4)	4.5 (2.0)	4.5 (2.1)	3.7 (2.3)	0.308
**PF**					
EI (kJ)	496 (285)	423 (196)	599 (277)	480 (274)	0.593
EI (%)	8.1 (3.4)	7.0 (3.7)	9.9 (4.3)	8.9 (4.1)	0.145
**UPF**					
EI (kJ)	2607 (682)	2956 (760)	3243 (822)	3230 (883)	0.003
EI (%)	44.9 (8.7)	55.3 (15.9)	52.8 (9.5)	54.9 (9.6)	0.009

Abbreviations: EI, energy intake; FFQ, food frequency questionnaire; kJ, kilojoules; MPF, minimally processed food; PCI, processed culinary ingredient; PF, processed food; SD, standard deviation; UPF, ultra-processed food; WDR, weighed diet record.

**Table 3:** Cross-classification of quartiles of daily energy intake from processed food categories by FFQ and WDR (*n* = 100).

	Correctly Classified ^1^	Correctly or Adjacently Classified ^2^	Correct Extremes ^3^	Grossly Misclassified ^4^
*Chance*	*25%*	*62.5%*	*12.5%*	*12.5%*
**MPF**				
EI (kJ)	23	70	15	9
EI (%)	33	72	19	7
**PCI**				
EI (kJ)	31	67	13	14
EI (%)	30	66	15	12
**PF**				
EI (kJ)	25	70	13	13
EI (%)	29	68	13	9
**UPF**				
EI (kJ)	29	73	18	6
EI (%)	32	74	20	4

^1^ Correctly classified = % of children with WDR and FFQ intakes in the same quartile; ^2^ correctly or adjacently classified = % of children with WDR and FFQ intakes in the same or adjacent quartiles; ^3^ correct extremes = % of children with WDR and FFQ intakes correctly classified to the lowest and highest quartiles; ^4^ grossly misclassified = % of children with WDR intakes in the highest quartile and FFQ intakes in the lowest quartile, or vice versa. Abbreviations: EI, energy intake; FFQ, food frequency questionnaire; kJ, kilojoules; MPF, minimally processed food; PCI, processed culinary ingredient; PF, processed food; UPF, ultra-processed food; WDR, weighed diet record.

**Table 4:** Test–retest reliability: Difference and intra-class correlation between daily energy intake estimates from processed food categories between both FFQ administrations (*n* = 99).

	FFQ1 Mean (SD)	FFQ2 Mean (SD)	Mean Difference (95% CI) between both FFQ Administrations	p-Value	95% Limits of Agreement	Intra-Class Correlation Coefficient
**MPF**						
EI (kJ)	2802 (971)	2564 (855)	−239 (−373, −104)	0.001	0 to 1108	0.73
EI (%)	39.1 (8.4)	37.9 (9.4)	−1.2 (−2.6, 0.2)	0.102	−15.6 to 13.2	0.67
**PCI**						
EI, kJ	44.2 (58.3)	36.9 (34.0)	−7.3 (−16.7, 2.2)	0.129	−102 to 87	0.51
EI, %	0.63 (0.74)	0.56 (0.55)	−0.07 (−0.18, 0.04)	0.196	−1.1 to 1.0	0.64
**PF**						
EI, kJ	243 (142)	240 (119)	−3 (−25, 19)	0.764	−223 to 217	0.65
EI, %	3.4 (1.9)	3.6 (1.7)	0.1 (−0.1, 0.4)	0.336	−2.6 to 2.9	0.70
**UPF**						
EI, kJ	4102 (1393)	4003 (1427)	−99 (−295, 97)	0.319	0 to 1868	0.76
EI, %	56.8 (8.6)	58.0 (9.3)	1.1 (−0.4, 2.6)	0.141	−14.0 to 16.3	0.64

Abbreviations: EI, energy intake; FFQ, food frequency questionnaire; kJ, kilojoules; MPF, minimally processed food; PCI, processed culinary ingredient; PF, processed food; UPF, ultra-processed food.

## Supplementary Materials

The following are available online at https://www.mdpi.com/2072-6643/11/6/1290/s1, Table S1: EAT5 Food Frequency Questionnaire

## Appendix A

**Table A1:** Difference in percentage weight (g) and percentage of energy (kJ) content by food processing, of ten randomly selected recipes from WDR ^1^.

	Processed Food Category			
Mean proportion of recipes	MPF	PCI	PF	UPF
By weight (g)	60.5	8.6	13.2	17.7
By energy content (kJ)	52.8	12.5	12.5	22
Mean difference between weight (g) and energy (kJ) content of recipes	7.7	−3.9	0.7	−4.3

^1^ The ten recipes were: chocolate chunk cookies, macaroni and pineapple bake, pizza base, ham sandwich, peanut butter and cheese sandwich, tomato soup, pork stir-fry, cinnamon pinwheel scones, coconut rice, and mince pie filling. Abbreviations: MPF, minimally processed food; PCI, processed culinary ingredient; PF, processed food; UPF, ultra-processed food; WDR, weighed diet record.
