# Supplementary material for: Relative Validity and Reproducibility of a Food Frequency Questionnaire to Assess Energy Intake from Minimally Processed and Ultra-Processed Foods in Young Children
Source: Nutrients. 2019 Jun 7;11(6):1290. doi: 10.3390/nu11061290 (PMC6627316; doi:10.3390/nu11061290)
Supplement: Supplementary file 1 [file nutrients-11-01290-s001.pdf]

5 year Food Frequency Questionnaire

To be administered to the child's main carer

EAT5 study number [| | |]

Interview date DD MM YY

Interviewer name ____________________

You will need:

- This questionnaire
- Ready reckoners 1 and 2
- Plate
- Bowl
- Mug
- Glass
- Measuring cup
- Measuring spoons
- Funnel
- Beans
- Rice

Instructions for the interviewer:

- Please check that the POI study number, the date, and your name are **recorded on the front page** before starting the questionnaire
- Ask each question in turn **as it is worded** in the questionnaire.
- Each food should be **entered only once** and **as separate items** where possible. For example, if they had custard and peaches for pudding, this should be entered as i) custard and ii) peaches – not as "puddings not yet described".
- **Mixed dishes should be entered separately** – so lasagne should be entered as appropriate amounts of "Other pasta (pasta only - not including sauce)", "Mince & patties (from beef or lamb)", and "White or cheese sauce", and "Cooked tomato (pasta sauce, canned tomatoes etc)".
- Room has been left at the bottom of each page to **make comments** that will help with interpreting the data – we would like you to put as much information as possible. For instance a child might have a slice of bread that is from a breadmaker which is much thicker than the standard toaster slice.

Choosing a frequency

- Enter **only one value for frequency** (that is, how often a week OR day, not both)
- Make sure there is a frequency for **every food** (i.e., use the "not eaten this month" column as appropriate)
- Use "Ready Reckoner 1" if parent says that the child has something **regularly but only for part of the week**, for example, "Beth has a banana twice a day during the week but not at all in the weekend". This will give you the single frequency option to use.
- Use "Ready Reckoner 2" for those **questions that include more than one food**, for example "white buns (not iced), crumpets". This will give you the single frequency option to use.

Estimating the amount eaten

- **For those foods that have a "standard" unit provided,** the parent needs to estimate how many of these units (or fractions of units such as ¼ or ½) the child would usually eat (1st example on the next page).
- **For those foods with units of ml,** you need to use the beans (larger foods) or rice (smaller foods) to estimate the volume they consume. Ask the parent to pour the beans or rice into the plate or cup (whichever is appropriate) to the level that their child would usually eat. You then pour this amount into the measuring cup and record the volume (2nd example on the next page).

		How often have they eaten this food this month? *Complete one frequency column only per food*										How much did they eat each time?	
**Example**	**Food**	Not eaten this month	Less than once a week	Once a week	2 times a week	3 times a week	4 times a week	5 times a week	6 times a week	Every day	If more than once a day – how many times a day	Units	How many [units] would they eat each time?
1	Instant noodles			✓								1 packet	¾
2	Cornflakes or rice bubbles							✓				140 ml	

Instructions for the participant:

- These questions ask how often and how much *child's name* has eaten certain foods or beverages **over the past month.**
- *Child's name* may sometimes be fed by a relative, friend, or someone else. If you know the type of food and approximate amount s/he has eaten at these times please include them.
- Please tell us what they **actually** ate and drank; we are interested in what children actually eat, not the perfect diet.
- All information is stored by study number and not by your name.

A. Bread, crackers and breakfast cereals

		How often have they eaten this food this month? *Complete one frequency column only per food*										How much did they eat each time?	
	**Food**	Not eaten this month	Less than once a week	Once a week	2 times a week	3 times a week	4 times a week	5 times a week	6 times a week	Every day	If more than once a day – how many times a day	Units	How many [units] would they eat each time?
1	White bread											1 toaster slice	
2	White buns (not iced), crumpets											1 small bun or 1 crumpet	
3	Wholemeal bread or bun											1 toaster slice or 1 small bun	
4	Wholegrain bread or bun											1 toaster slice or 1 small bun	
5	Crackers (wheat, rice or corn-based)											1 cracker	
6	Rice cakes or rice wheels*											1 rice cake or 17 rice wheels	
7	Cruskits or crispbreads											1 cruskit	

Comments?

* Rice wheel = tiny kids' size rice cake (~ 50 cent coin in diameter)

* Rice cake = puffed rice crackers the size of a large cookie

		How often have they eaten this food this month? *Complete one frequency column only per food*										How much did they eat each time?	
	**Food**	Not eaten this month	Less than once a week	Once a week	2 times a week	3 times a week	4 times a week	5 times a week	6 times a week	Every day	If more than once a day – how many times a day	Units	How many [units] would they eat each time?
8	Weet-bix											1 weetbix	
9	Fruity-bix or similar											_______ ml	
10	Porridge											_______ ml	
11	Cornflakes or rice bubbles											_______ ml	
12	Cocopops, honey puffs or puffed wheat cereal											_______ ml	
13	Nutrigrain, Milo cereal or similar											_______ ml	
14	Muesli and "light" muesli (e.g. "Light and Tasty, Light and Right)"											_______ ml	
15	Other breakfast cereal (record name of main one): _______________											_______ ml	

Comments?

B. Rice and pasta

		How often have they eaten this food this month? *Complete one frequency column only per food*										How much did they eat each time?	
	**Food**	Not eaten this month	Less than once a week	Once a week	2 times a week	3 times a week	4 times a week	5 times a week	6 times a week	Every day	If more than once a day – how many times a day	Units	How many [units] would they eat each time?
16	White rice											_______ ml	
17	Brown rice											_______ ml	
18	Instant noodles											1 packet	
19	Canned spaghetti											1 medium can (420g)	
20	Other pasta (pasta only - not including sauce)											_______ ml	
21	Pizza (not takeaway) – base only											1 slice	

Comments?

C. Fruit

22 How often has your child had fruit in the past month?

_____ less than once a week OR _____ times a week OR _____ times a day

	Food	How often have they eaten this food this month? *Complete one frequency column only per food*										How much did they eat each time?	
		Not eaten this month	Less than once a week	Once a week	2 times a week	3 times a week	4 times a week	5 times a week	6 times a week	Every day	If more than once a day – how many times a day	Units	How many [units] would they eat each time?
23	Banana											1 banana*	
24	Apples (fresh or canned)											1 apple	
25	Apricots, plums or peaches (fresh or canned)											1 apricot or 1 plum, or ½ peach	
26	Pears (fresh or canned)											1 pear	
27	Raisins or sultanas											1 small box (12g)	
28	Dried apricots or prunes											1 prune	
29	Oranges, mandarins											1 orange or 2 mandarins	

Comments?

*2 bobby bananas = 1 medium banana

	Food	How often have they eaten this food this month? *Complete one frequency column only per food*										How much did they eat each time?	
		Not eaten this month	Less than once a week	Once a week	2 times a week	3 times a week	4 times a week	5 times a week	6 times a week	Every day	If more than once a day – how many times a day	Units	How many [units] would they eat each time?
30	Kiwifruit											1 kiwifruit	
31	Green grapes											1 grape	
32	Black or red grapes											1 grape	
33	Berries or cherries (fresh or frozen)											_______ ml	
34	Avocado											1 avocado	
35	Rhubarb											_______ ml	
36	Other fruit											_______ ml	

Comments?

D. Vegetables

37 How often has your child had vegetables in the past month?

_____ less than once a week OR _____ times a week OR _____ times a day

		How often have they eaten this food this month? *Complete one frequency column only per food*										How much did they eat each time?	
	**Food**	Not eaten this month	Less than once a week	Once a week	2 times a week	3 times a week	4 times a week	5 times a week	6 times a week	Every day	If more than once a day – how many times a day	Units	How many [units] would they eat each time?
38	Potato salad or other potato eaten cold											_____ ml	
39	Potato or kumara (boiled, baked, microwaved, mashed) eaten warm or hot											_____ ml	
40	Hot chips, potato shapes, roast potato or kumara cooked at home eaten warm or hot											_____ ml	
41	Yams											_____ ml	
42	Carrot											_____ ml	
43	Pumpkin											_____ ml	
44	Green peas											_____ ml	
45	Green beans											_____ ml	

Comments?

		How often have they eaten this food this month? *Complete one frequency column only per food*										How much did they eat each time?	
	**Food**	Not eaten this month	Less than once a week	Once a week	2 times a week	3 times a week	4 times a week	5 times a week	6 times a week	Every day	If more than once a day – how many times a day	Units	How many [units] would they eat each time?
46	Sweet corn											_____ ml	
47	Broccoli											_____ ml	
48	Cauliflower											_____ ml	
49	Capsicum (peppers)											_____ ml	
50	Red cabbage											_____ ml	
51	Green cabbage											_____ ml	
52	Spinach or silverbeet											_____ ml	
53	Lettuce or salad leaves											_____ ml	
54	Cucumber											1 slice	
55	Raw tomato											1 tomato	
56	Cooked tomato (pasta sauce, canned tomatoes, tomato sauce)											_____ ml	
57	Leeks											_____ ml	
58	Other vegetables: _____											_____ ml	

Comments?

E. Meat, chicken, fish, eggs, beans

		How often have they eaten this food this month? *Complete one frequency column only per food*										How much did they eat each time?	
	**Food**	Not eaten this month	Less than once a week	Once a week	2 times a week	3 times a week	4 times a week	5 times a week	6 times a week	Every day	If more than once a day – how many times a day	Units	How many [units] would they eat each time?
59	Chicken nuggets or shapes											1 nugget	
60	Fish fingers or shapes											1 fish finger	
61	Battered or crumbed fish											1 fillet	
62	Other chicken (eg. roast, stir-fry, BBQ)											1 drumstick	
63	Other fish (eg. canned, pan-fried)											_______ ml	
64	Sausages, saveloys (including vegetarian)											1 sausage	
65	Ham, bacon, luncheon, salami											1 slice	
66	Meat pies											1 pie	
67	Sausage rolls											1 roll	
68	Mince & patties (from beef or lamb)											_______ ml	

Comments?

		How often have they eaten this food this month? *Complete one frequency column only per food*										How much did they eat each time?	
	**Food**	Not eaten this month	Less than once a week	Once a week	2 times a week	3 times a week	4 times a week	5 times a week	6 times a week	Every day	If more than once a day – how many times a day	Units	How many [units] would they eat each time?
69	Steak, chops or roast (beef or lamb)											_______ ml	
70	Pork and other meat											_______ ml	
71	Eggs											1 egg	
72	Hummus (chickpea dip)											_______ ml	
73	Baked beans											1 medium can (420g)	
74	Canned or home cooked beans, chickpeas or lentils											_______ ml	
75	Nuts (any sort but not peanut butter)											_______ ml	

Comments?

F. Spreads

	Food	How often have they eaten this food this month? *Complete one frequency column only per food*										How much did they eat each time?	
		Not eaten this month	Less than once a week	Once a week	2 times a week	3 times a week	4 times a week	5 times a week	6 times a week	Every day	If more than once a day – how many times a day	Units	How many [units] would they eat each time?
76	Jam or honey											_______ ml	
77	Marmite or Vegemite											_______ ml	
78	Peanut butter											_______ ml	
79	Nutella											_______ ml	

Comments?

G. Cakes, biscuits, snacks

	Food	How often have they eaten this food this month? *Complete one frequency column only per food*										How much did they eat each time?	
		Not eaten this month	Less than once a week	Once a week	2 times a week	3 times a week	4 times a week	5 times a week	6 times a week	Every day	If more than once a day – how many times a day	Units	How many [units] would they eat each time?
80	Biscuits – chocolate coated											1 biscuit	
81	Biscuits – other											1 biscuit	
82	Cakes or slices											1 large raisin box	
83	Muffins or scones											1 medium muffin	
84	Croissant, sweet buns, iced buns, pastries											1 item	
85	Fruit bread, currant buns											1 slice	
86	Chocolate											1 square	
87	Lollies											1 lolly	
88	Crisps, corn chips, corn snacks (e.g. Cheezels)											1 snack packet (18g)	
89	Muesli, nut, cereal or puffed rice bars											1 bar	
90	Fruit leather, fruit strings, fruit roll-ups											_______ ml	

Comments?

H. Milk and dairy products

	Food	How often have they eaten this food this month? *Complete one frequency column only per food*										How much did they eat each time?	
		Not eaten this month	Less than once a week	Once a week	2 times a week	3 times a week	4 times a week	5 times a week	6 times a week	Every day	If more than once a day – how many times a day	Units	How many [units] would they eat each time?
91	Flavoured milk (including Milo, Quick, Drinking chocolate, Up-and-Go)											_______ ml	
92	Low-fat cows milk (green, lite blue, yellow-top) **as a drink**											_______ ml	
93	Low-fat cows milk **on cereal or other food** (not custard or sauces)											_______ ml	
94	Cows milk (blue, silver-top) **as a drink**											_______ ml	
95	Cows milk **on cereal or other food** (not custard or sauces)											_______ ml	
96	Soy milk **as a drink**											_______ ml	
97	Soy milk **on cereal or other food** (including custard or sauces)											_______ ml	
98	Other milk (goat, rice) **as a drink**											_______ ml	
99	Other milk (goat, rice) **on cereal or other food**											_______ ml	

Comments?

	Food	How often have they eaten this food this month? *Complete one frequency column only per food*										How much did they eat each time?	
		Not eaten this month	Less than once a week	Once a week	2 times a week	3 times a week	4 times a week	5 times a week	6 times a week	Every day	If more than once a day – how many times a day	Units	How many [units] would they eat each time?
100	Cheese (including in recipes)											_______ ml	
101	Yoghurt or dairy food											_______ ml	
102	White sauce or cheese sauce											_______ ml	
103	Butter (not in baking)											_______ ml	
104	Margarine (not in baking)											_______ ml	
105	Cream or sour cream											_______ ml	

Comments?

I. Puddings

		How often have they eaten this food this month? *Complete one frequency column only per food*										How much did they eat each time?	
	**Food**	Not eaten this month	Less than once a week	Once a week	2 times a week	3 times a week	4 times a week	5 times a week	6 times a week	Every day	If more than once a day – how many times a day	Units	How many [units] would they eat each time?
106	Ice cream											_______ ml	
107	Custard and other milk puddings											_______ ml	
108	Puddings not yet described											_______ ml	

Comments?

J. Drinks

		How often have they eaten this food this month? *Complete one frequency column only per food*										How much did they eat each time?	
	**Food**	Not eaten this month	Less than once a week	Once a week	2 times a week	3 times a week	4 times a week	5 times a week	6 times a week	Every day	If more than once a day – how many times a day	Units	How many [units] would they eat each time?
109	100% orange juice (freshly squeezed or similar)											_______ ml	
110	Other fruit juice ("Fresh up", "Just Juice")											_______ ml	
111	Fruit drinks, Ribena, cordial, sachets											_______ ml	
112	Regular fizzy drinks (lemonade, coke)											_______ ml	
113	Diet fizzy drinks (lemonade, coke)											_______ ml	
114	Tea (not herbal)											_______ ml	
115	Coffee											_______ ml	

Comments?

K. Takeaways

		How often have they eaten this food this month? *Complete one frequency column only per food*										How much did they eat each time?	
	Food	Not eaten this month	Less than once a week	Once a week	2 times a week	3 times a week	4 times a week	5 times a week	6 times a week	Every day	If more than once a day – how many times a day	Units	How many [units] would they eat each time?
116	Chips from a takeaway shop or fast food restaurant (KFC, McDonalds, Burger King)											_______ ml	
117	Hotdog, fish, sausage from a takeaway shop											1 item	
118	Burgers from a takeaway shop or fast food restaurant (KFC, McDonalds, Burger King)											1 burger	
119	Other item from a takeaway shop or fast food restaurant (KFC, McDonalds, Burger King (please describe)											1 item	
120	Ready to eat pizza (takeaway shop or supermarket)											1 slice	
121	KFC or other fried chicken											1 piece	
122	Subway sandwich											1 6" roll	
123	Kebabs or wraps (bought)											1 wrap	
124	Sushi (bought)											1 piece	
125	Chinese, Thai, or Indian meal or similar (bought)											_______ ml	

Comments?

126 On average over the past 4 weeks, how many meals **per week** were given to your child by someone other than yourself? _____________ meals

127 If more than 0 meals, how many of these meals have you been able to include in this questionnaire?

☐ None ☐ Some ☐ Most ☐ All

Thank you for completing this questionnaire

UNIVERSITY of OTAGO
Te Whare Wānanga o Otāgo
NEW ZEALAND
SAPERE AUDE

Department of Human Nutrition and
Department of Medicine
University of Otago
P. O. Box 56, Dunedin 9054
Email:
Phone:

eat5
